# Comparison of radiotherapy combined with nimotuzumab vs. chemoradiotherapy for locally recurrent nasopharyngeal carcinoma

**DOI:** 10.1186/s12885-021-08995-y

**Published:** 2021-11-25

**Authors:** Jing-Feng Zong, Qian-Dong Liang, Qiong-Jiao Lu, Yu-Hong Liu, Han-Chuan Xu, Bi-Juan Chen, Qiao-Juan Guo, Yun Xu, Cai-Rong Hu, Jian-Ji Pan, Shao-Jun Lin

**Affiliations:** 1 Department of Radiation Oncology, Fujian Medical University Cancer Hospital, Fujian Cancer Hospital, No. 420 Fuma Road, Fuzhou, 350014 Fujian China; 2 Fujian Key Laboratory of Translational Cancer Medicine, Fuzhou, Fujian China; 3 Key Laboratory of Systems Biomedicine (Ministry of Education), Shanghai Center for Systems Biomedicine, Shanghai Jiao Tong University, Shanghai, China

**Keywords:** Locally recurrent nasopharyngeal carcinoma, Radiotherapy, Nimotuzumab, Chemoradiotherapy, Toxicity

## Abstract

**Background:**

The present study compared the effectiveness and toxicity of two treatment modalities, namely radiotherapy combined with nimotuzumab (N) and chemoradiotherapy (CRT) in patients with locally recurrent nasopharyngeal carcinoma (LR-NPC).

**Methods:**

Patients with LR-NPC who were treated with radiotherapy were retrospectively enrolled from January 2015 to December 2018. The treatment included radiotherapy combined with N or platinum-based induction chemotherapy and/or concurrent chemotherapy. The comparison of survival and toxicity between the two treatment modalities was evaluated using the log-rank and chi-squared tests. Overall survival (OS) was the primary endpoint.

**Results:**

A total of 87 patients were included, of whom 32 and 55 were divided into the N group and the CRT group, respectively. No significant differences were noted in the survival rate between the N and the CRT groups (4-year OS rates, 37.1% vs. 40.7%, respectively; *P* = 0.735). Mild to moderate acute complications were common during the radiation period and mainly included mucositis and xerostomia. The majority of the acute toxic reactions were tolerated well. A total of 48 patients (55.2%) demonstrated late radiation injuries of grade ≥ 3, including 12 patients (37.5%) in the N group and 36 patients (66.5%) in the CRT group. The CRT group exhibited significantly higher incidence of severe late radiation injuries compared with that of the N group (*P* = 0.011).

**Conclusion:**

Radiotherapy combined with N did not appear to enhance treatment efficacy compared with CRT in patients with LR-NPC. However, radiotherapy combined with N may be superior to CRT due to its lower incidence of acute and late toxicities. Further studies are required to confirm the current findings.

## Background

Nasopharyngeal carcinoma (NPC) is one of the most common head and neck tumors, with unique epidemiological features and pathological characteristics. Nearly half of the patients with NPC in the world are new cases diagnosed in China. Approximately 95% of these cases are non-keratinizing carcinomas (including differentiated and undifferentiated types) [1]. The primary treatment for NPC is radiotherapy (RT). Due to intensity-modulated radiation therapy (IMRT), approximately 8–10% of patients develop residual or local/regional recurrence following initial RT [2].

Locally recurrent NPC (LR-NPC) is mainly treated with salvage therapy, such as nasopharyngectomy and re-irradiation, which can improve the long-term survival rate of certain patients [3]. The early recurrent form of the disease exhibits confined lesions and the 5-year survival rates with salvage surgical resection can reach 50–77.1% [4–6]. However, approximately 60% of LR-NPC cases are clinically advanced, invasive and difficult to be removed by surgery. Therefore, external radiation therapy is still the main form of treatment for this disease [2, 7].

Despite the lack of conclusive evidence, clinicians often administer concurrent chemotherapy to reduce the tumor volume and improve radiosensitivity prior to or during re-irradiation. The combination of radiation and chemotherapy may exacerbate the treatment toxicity and counteract the benefits of chemotherapy. In addition, when combined with chemotherapy, patients are less tolerant to re-irradiation. It has been reported that the incidence of grade 5 toxicity for re-irradiation can reach 33% [8]. Therefore, the development of more effective and less toxic treatment options is imperative.

Epidermal growth factor (EGF) is the expression product of the oncogene cerbB1 and has been shown to be associated with poor tumor prognosis in numerous studies [9–11]. Nimotuzumab (N) is a humanized anti-EGFR monoclonal antibody that exhibits antitumor and enhanced radiosensitivity properties [12, 13]. Although N has been shown to be effective in locally advanced NPC [12, 14], a limited number of studies have examined LR-NPC to date. The aim of the present study was to evaluate the efficacy and safety of RT combined with N in patients with LR-NPC.

## Materials and methods

### Patient selection criteria

The present retrospective study was approved by the Ethics Committee of Fujian Cancer Hospital. All patients with LR-NPC, whose clinical records were selected, signed the written informed consent prior to treatment, and were treated from January 2015 to December 2018. Local recurrence was defined as tumor relapse occurring at least 6 months after initial radiotherapy. Pathological and histological evidence is required for nasopharyngeal and skull base recurrence; however, if pathological and histological evidence cannot be obtained for the latter, evidence of imaging progression is required. The following additional inclusion criteria were used: age range 18–70 years, Karnofsky performance score (KPS) ≥70, lack of distant metastases or other malignancies, normal hematological examination results and normal hepatic and renal function. All patients included in the analysis were restaged by two radiotherapists of intermediate or higher rank according to the American Joint Committee on Cancer and Union for International Cancer Control (8th edition) TNM staging of NPC.

### Treatment

#### Radiation therapy

All patients were treated with IMRT methodological techniques. Head and neck fixation was performed using a thermoplastic mask and a base frame. The computer tomography/magnetic resonance imaging (CT/MRI) fusion technique was used to outline the gross tumor volume (GTV) and the clinical target volume (CTV) and to identify the organs at risk on each CT image layer. GTV was defined as tumor lesions noted on clinical examination, endoscopy and CT/MRI/positron emission tomography-computer tomography (PET-CT), including nasopharyngeal tumors, retropharyngeal lymph nodes and positive lymph nodes in the neck. Positive lymph nodes in the neck were defined as lymph nodes with a maximum transverse diameter > 1 cm, or enlarged lymph nodes indicating central necrosis, distribution of at least 3 lymph nodes of critical size in clusters and PET-CT positive lymph nodes. CTV was defined as GTV and subclinical lesions, including GTV and 6–10 mm outside of GTV. Planning target volume (PTV) was defined as a certain safety boundary (3 mm) placed outside of GTV/CTV to compensate for positional errors and intrinsic organ motion. In case GTV or CTV were adjacent to important organs, the range of the safety boundary could be reduced as appropriate. The GTV-PTV prescription dose range was 60–66 Gy, whereas the radiation rate was 2.0 Gy/dose and radiation frequency range was 30–33. A second set of parameters were used that included the following: CTV-PTV prescription dose range was 50–55 Gy, whereas the radiation rate was 1.67 Gy/dose and radiation frequency range was 30–33. The organs at risk (OARs) included the following: brainstem, spinal cord, pituitary gland, optic chiasm, eye, lens, optic nerve, temporal lobe, hippocampus, temporomandibular joint, mandible and parotid gland. As a general rule, the dose constraints of the OARs limit are < 70% of the tolerance dose, which refers to a serve complication rate of 5 within 5 years of radiotherapy (TD5/5). The optic chiasm, brain stem and spinal cord had the highest priority, followed by the GTV, then other less critical OARs, such as temporal lobes and optic nerves. No dose limitations were set for the parotid glands as all the patients had already received high doses to those structures in their conventional RT. A conventional segmentation pattern was used, once a day, 5 times a week.

#### Chemotherapy

Standardized regimens for the treatment of LR-NPC were not available when the patients were treated. Therefore, the chemotherapy administered in our center was based on the preference of the clinical physicians and the patients. The most commonly used chemotherapy regimen was based on platinum (cisplatin 80 mg/m^2^ over 3 days or nedaplatin 80–100 mg/m^2^). The induction chemotherapy included gemcitabine (1000 mg/m^2^ on days 1 and 8) + platinum, docetaxel (60 mg/m^2^ on day 1) + 5-fluorouracil (600 mg/m^2^; continuous administration for 96 h IV drip) + platinum, paclitaxel (135–175 mg/m^2^, day 1) + platinum, 5-fluorouracil (800–1000 mg/m^2^; continuous administration for 120 h IV drip) + platinum and other regimens. Concurrent chemotherapy was administered with single-agent platinum every 3 weeks. Induction chemotherapy was administered for 2–4 cycles and concurrent chemotherapy for 2 cycles.

#### Targeted therapy

Existing guidelines for LR-NPC do not provide recommendations regarding targeted therapy. The patients of the present study treated with N were derived from a phase II clinical trial (NCT03666221) conducted in our institution. The regimen was as follows: N was initiated 1 week prior to radiation therapy and was thereafter administered once a week at 200 mg for 8 times concurrently with RT. Cardiac monitoring was performed during the drug administration. Chemotherapy was not used prior to, during or following RT.

### Follow-up

By using telephone communication or clinical notes, survival, tumor status and treatment toxicities were documented. Following completion of the treatment, the patients were followed up every 3 months for 2 years, every 6 months for the next 3 years and every 12 months thereafter. The follow-up visits included a complete medical history, systematic physical examination, plasma Epstein-Barr virus (EBV) DNA, nasopharyngeal endoscopy, nasopharyngeal and neck MRI (once every 6 months), chest radiography and abdominal ultrasonography. A whole-body bone scintigraphy was carried out if necessary. Tumor responses were evaluated 3 months following completion of radiotherapy, according to the Response Evaluation Criteria in Solid Tumors, version 1.1. Acute toxicity and late radiation injuries were evaluated according to the Radiation Therapy Oncology Group toxicity evaluation criteria and the Common Terminology Criteria for Adverse Events v4.0. Acute toxicity was defined from day 1 to day 90 following treatment initiation. Late radiation injuries were defined as toxic reactions that occurred 90 days following the completion of the patient radiation therapy. The follow-up time was defined as the time from diagnosis of NPC until the date of death or the date of the last follow-up. The final follow-up time was performed in September 2020.

### Statistical analysis

Overall survival (OS) was defined as the time from day 1 following completion of treatment to the last examination or patient death. Progression-free survival (PFS) was defined as the time from day 1 following completion of treatment to the onset of local or regional recurrence, distant metastasis, or death from any cause. Local-regional failure-free survival (LRFFS) was defined as the time from day 1 following completion of treatment to the onset of local or regional recurrence. Distant metastasis-free survival (DMFS) was defined as the time from day 1 following completion of treatment to the appearance of distant metastases. Clinical baseline characteristics of patients were described and differences between the N and chemoradiotherapy (CRT) groups were compared by the chi-squared tests for categorical variables. OS, PFS, LRFFS and DMFS were estimated using Kaplan-Meier survival analysis and compared between the two groups using a log-rank test. The chi-square test (or Fisher’s exact test, if indicated) was also used to compare the pattern of failure and cause of mortality between the two treatment groups. Potential prognostic factors included clinical characteristics (gender, age and hemoglobin concentration), disease status (recurrent T classification, lymph node recurrence, GTV-T volume), time to recurrence, tumor response following RT, plasma EBV DNA prior to and following RT and treatment modality (N or chemotherapy). Univariate and multivariate survival prognostic analyses were performed using the Cox proportional hazards model. Factor analysis associated with severe late radiation injuries was performed using the chi-squared test and logistic regression analysis. All statistical analyses were performed using Statistical Product and Service Solutions, version 26.0 (IBM Corp.). All factors were tested using two-sided tests. *P* ≤ 0.05 was considered to indicate statistically significant differences.

## Results

### Patient characteristics

**Fig. 1:**
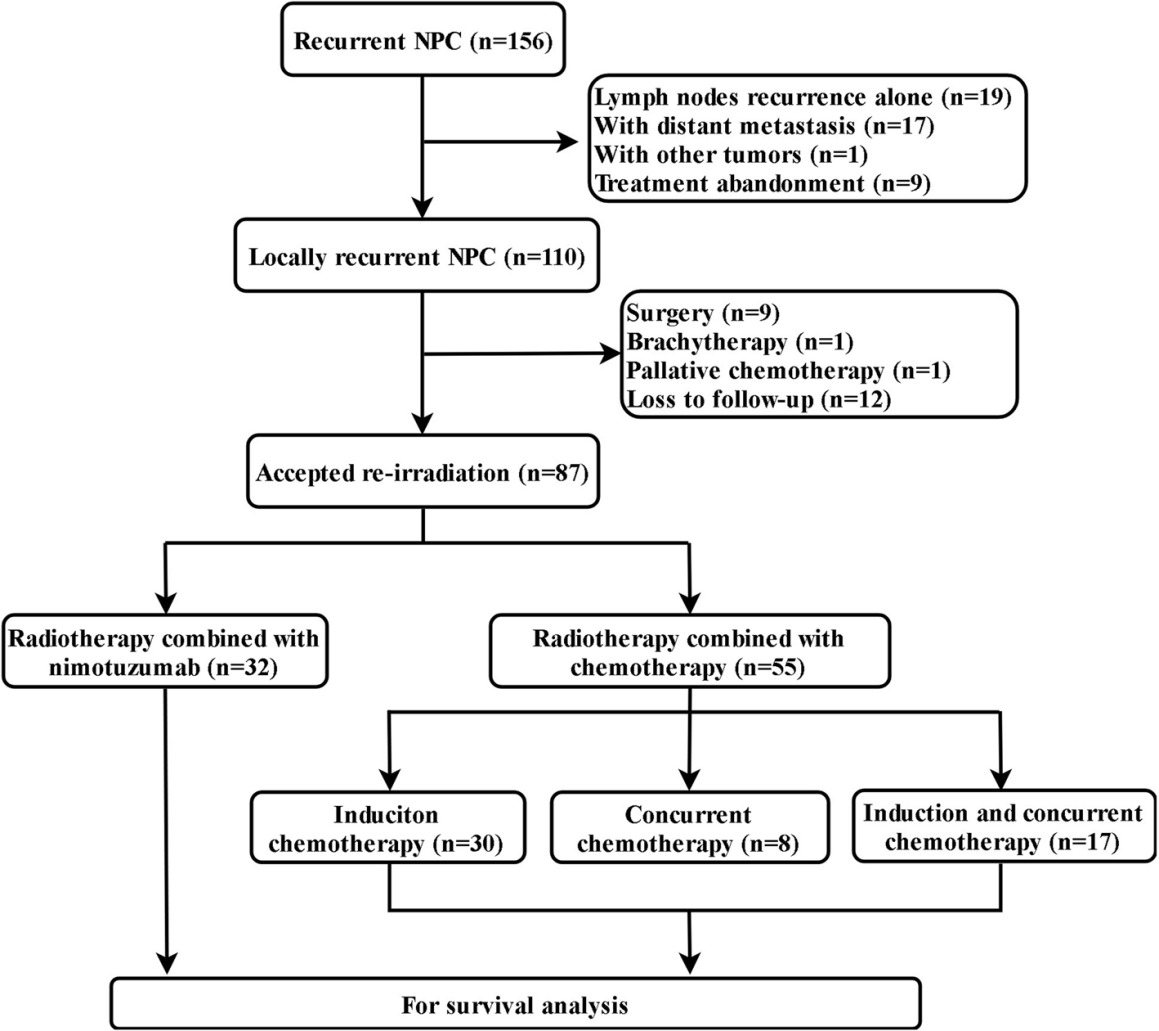
Flowchart of study patient inclusion. NPC, nasopharyngeal carcinoma

A total of 156 patients with recurrent NPC were treated at our center from January 2015 to December 2018 and 69 cases were excluded, including 19 cases with single lymph node regional recurrence, 17 cases with distant metastases, 1 case with other malignant tumors, 9 cases who did not complete treatment, 11 cases who did not receive IMRT treatment (including 9 cases treated with surgery, 1 case treated with brachytherapy and 1 case with palliative chemotherapy) and 12 cases who were lost to follow-up following treatment (Fig. 1). The exclusion of these patients was performed to ensure the accuracy and authenticity of the data.

The remaining 87 patients with LR-NPC were included in the analysis, of whom 69 (76.7%) were men and 21 (23.3%) were women. A total of 32 (36.8%) patients received RT combined with N and were included in the N group, whereas 55 (63.2%) received CRT and were included in the CRT group. In the CRT group, 30 cases (54.5%) received induction chemotherapy, whereas 8 cases (14.5%) received concurrent chemotherapy and 17 cases (30.9%) both induction chemotherapy and concurrent chemotherapy. A total of 4 cases (all in CRT group) were treated with a re-irradiation dose of 50 Gy/25 fraction due to poor RT tolerance. The analysis of the clinical baseline characteristics of the patients in each group demonstrated no significant differences by gender, age, histology, recurrent T classification, recurrent N classification and recurrent clinical stage between the N and the CRT groups (*P* > 0.05). The baseline characteristics of each group are detailed in Table 1.

**Table 1:** Baseline characteristics of patients in the N group and CRT group

Characteristic	N group (*N* = 32)	CRT group (*N* = 55)	P
	No. (%)	No. (%)	
Gender			0.886
Male	24 (75.0%)	42 (76.4%)	
Female	8 (25.0%)	13 (23.6%)	
Age (years)			0.193
< 50	18 (56.2%)	23 (41.8%)	
≥ 50	14 (43.8%)	32 (58.2%)	
KPS score			0.814
70–80	11 (34.4%)	17 (30.9%)	
90–100	21 (65.6%)	38 (69.1%)	
Histology			0.439
Keratinizing (WHO type I)	1 (3.1%)	1 (1.8%)	
Non-keratinizing differentiated (WHO type II)	10 (31.3%)	11 (20.0%)	
Non-keratinizing undifferentiated (WHO type III)	21 (65.6%)	43 (78.2%)	
Recurrent T classification			0.205
T1	1 (3.1%)	6 (10.9%)	
T2	2 (6.3%)	5 (9.1%)	
T3	16 (50.0%)	16 (29.1%)	
T4	13 (40.6%)	28 (50.9%)	
Recurrent N classification			0.557
N0	24 (75.0%)	34 (61.9%)	
N1	5 (15.6%)	15 (27.3%)	
N2	2 (6.3%)	5 (9.0%)	
N3	1 (3.1%)	1 (1.8%)	
Recurrent Clinical Stage			0.416
I	1 (3.1%)	2 (3.6%)	
II	2 (6.2%)	8 (14.6%)	
III	15 (46.9%)	17 (30.9%)	
IVa	14 (43.8%)	28 (50.9%)	

N group, radiotherapy combined with nimotuzumab group; CRT group, chemoradiotherapy group; KPS, Karnofsky performance status; WHO, World Health Organization. The *P*-value was calculated using the χ2-test or Fisher’s exact test

### Efficacy

**Fig. 2:**
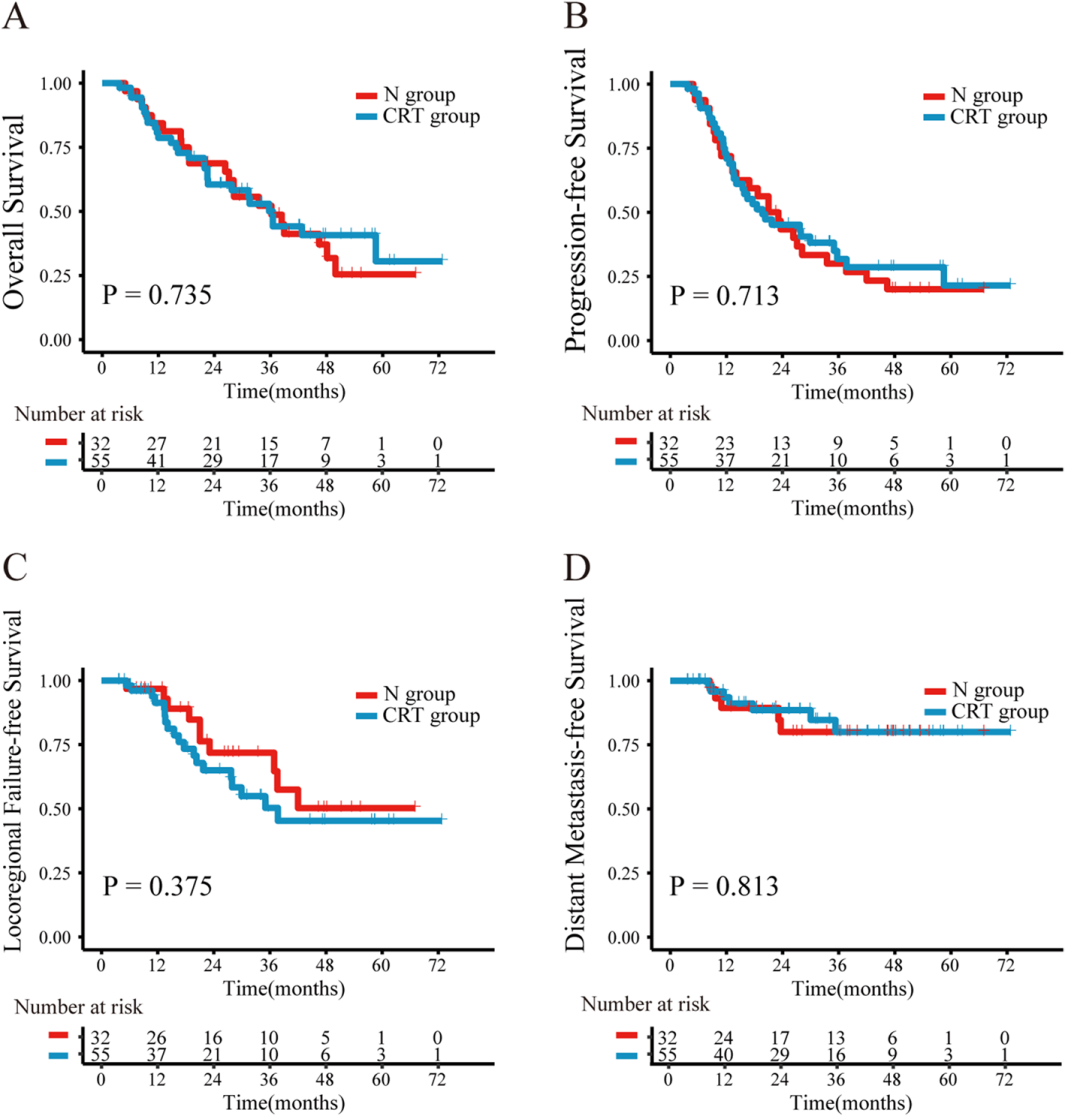
Kaplan-Meier curves of overall survival (**A**), progression-free survival (**B**), locoregional failure-free survival (**C**), and distant metastasis-free survival (**D**), according to radiotherapy combined with nimotuzumab or chemotherapy treatment in the 87 patients. *P* values were calculated with the log-rank test

All 87 patients completed therapy (including decreasing dose radiotherapy), with a median follow-up time of 47.8 months (3.7–72.8 months) and a median survival time of 36.2 months for the entire group. The 4-year OS of the N and the CRT groups were 37.1 and 40.7% (*P* = 0.735), respectively, whereas the 4-year PFS of the N and the CRT groups were 20.0 and 28.6%, respectively (*P* = 0.713). The 4-year LRFFS of the N and the CRT groups were 50.3 and 45.3%, respectively (*P* = 0.375), whereas the 4-year DMFS of both groups (N and CRT) was 80.0% (*P* = 0.813). No significant differences were noted between the two groups in terms of OS, PFS, LRFFS and DMFS. The survival curves are shown in Fig. 2. With respect to tumor response, there were 15 cases (46.9%) with complete response (CR) plus partial response (PR) in the N group, while there were 24 cases (43.6%) with CR plus PR in the CRT group (*P* = 0.770).

### Patterns of failure and causes of death

A total of 12 cases (13.7%) out of the total number of patients exhibited distant metastasis from the end of RT to the cut-off time point of the follow-up period. The median time period to metastasis was 12 months (range, 8.1–35.3 months). A total of 5 cases (15.6%) with metastases were noted in the N group, including 1 case of bone metastasis, 2 cases of lung metastasis, 1 case of liver metastasis and 1 case of distal lymph node metastasis. A total of 7 cases with metastases (12.7%) were noted in the CRT group, including 3 cases of bone metastasis, 2 cases of lung metastasis and 2 cases of distal lymph node metastasis. A second local or regional recurrence occurred in 29 cases (33.3%), with a median recurrence time of 18.7 months (range 5.2–42 months), of which 10 cases (31.3%) were noted in the N group and 19 cases (34.5%) in the CRT group. At the time of the analysis, 49 (56.3%) deaths occurred, of which 21 (65.6%) were noted in the N group and 28 (50.9%) in the CRT group. The most common cause of death was radiation therapy-associated complications, followed by tumor progression. A total of 26 cases (29.9%) did not survive due to RT-associated complications, of which 8 cases (25.0%) were from the N group and 18 cases (32.7%) were from the CRT group. A total of 15 cases (17.2%) did not survive due to tumor progression, of which 8 cases (25.0%) were from the N group and 7 cases (12.7%) were from the CRT group. The failure patterns and causes of death are shown in Table 2 and no significant differences were observed between the two groups with regard to failure patterns and causes of death (*P* > 0.05).

**Table 2:** Pattern of failure and cause of death

Variable	No. of patients (%)		
Pattern of failure	N Group	CRT Group	P
Distant metastasis	5^a^ (15.6%)	7^b^ (12.7%)	0.753
Local and/or regional failures	10^a^ (31.3%)	19^b^ (34.5%)	0.753
Local failures alone	3 (9.4%)	9 (16.4%)	0.523
Regional failures alone	1 (3.1%)	1 (1.8%)	1.000
Local and regional failures	6 (18.8%)	9 (16.4%)	0.776
Distant + local/regional failures	2 (6.3%)	5 (9.0%)	1.000
Total	13 (43.8%)	21 (38.2%)	0.822
**Cause of death**			
Tumor progression	8 (25.0%)	7 (12.7%)	0.144
Distant metastasis	2 (6.3%)	1 (1.8%)	0.552
Local or regional failure	5 (15.6%)	2 (3.6%)	0.095
Distant + local/regional failures	1 (3.1%)	4 (7.3%)	0.648
Radiation-related complications	8 (25.0%)	18 (32.7%)	0.448
Organ failure caused by tumor	1 (3.1%)	1 (1.8%)	1.000
No cancer causes	4 (12.5%)	1 (1.8%)	0.059
Unknown causes	0 (0.0%)	1 (1.8%)	1.000
Total	21 (65.6%)	28 (50.9%)	0.182

^a^ The number includes the two patients with both distant and local/regional failures; ^b^ The number includes the five patients with both distant and local/regional failures

### Toxicity

All patients completed the established RT schedule. Mild to moderate acute toxic reactions, including mucositis and xerostomia, were common during RT and the majority of the acute toxicities were considered as tolerable. During treatment, mild (grade 0–1) hematological toxic reactions were reported in group N. The proportion and degree of hepatotoxicity, weight loss, dermatitis, nausea and oral mucositis were significantly lower in the N group than those noted in the CRT group (Table 3).

**Table 3:** Acute toxicities of N group and CRT group

Toxicities	N group	CRT group	P
Leukopenia			< 0.001
G0	25 (78.1%)	16 (29.1%)	
G1	7 (21.9%)	17 (30.9%)	
G2	0 (0.0%)	14 (25.5%)	
G3	0 (0.0%)	6 (10.9%)	
G4	0 (0.0%)	2 (3.6%)	
Neutropenia			< 0.001
G0	30 (93.7%)	22 (40.0%)	
G1	2 (6.3%)	14 (25.5%)	
G2	0 (0.0%)	12 (21.8%)	
G3	0 (0.0%)	5 (9.1%)	
G4	0 (0.0%)	2 (3.6%)	
Anemia			0.085
G0	29 (90.7%)	37 (67.3%)	
G1	1 (3.1%)	10 (18.2%)	
G2	1 (3.1%)	6 (10.9%)	
G3	1 (3.1%)	2 (3.6%)	
Thrombocytopenia			0.082
G0	31 (96.9%)	42 (76.4%)	
G1	1 (3.1%)	5 (9.1%)	
G2	0 (0.0%)	6 (10.9%)	
G3	0 (0.0%)	2 (3.6%)	
Hepatoxicity			0.009
G0	28 (87.5%)	30 (54.6%)	
G1	4 (12.5%)	13 (23.6%)	
G2	0 (0.0%)	11 (20.0%)	
G3	0 (0.0%)	1 (1.8%)	
Weight Loss			0.047
G0	29 (90.6%)	40 (72.7%)	
G1	3 (9.4%)	15 (27.3%)	
Salivary glands injury			0.538
G0	3 (9.4%)	2 (3.6%)	
G1	27 (84.3%)	49 (89.1%)	
G2	2 (6.3%)	4 (7.3%)	
Dermatitis			0.005
G0	12 (37.5%)	4 (7.3%)	
G1	17 (53.1%)	45 (81.8%)	
G2	3 (9.4%)	5 (9.1%)	
G3	0 (0.0%)	1 (1.8%)	
Nausea			< 0.001
G0	28 (87.5%)	21 (38.2%)	
G1	3 (9.4%)	29 (52.7%)	
G2	1 (3.1%)	5 (9.1%)	
Vomiting			0.612
G0	28 (87.5%)	45 (81.8%)	
G1	3 (9.4%)	4 (7.3%)	
G2	1 (3.1%)	5 (9.1%)	
G3	0 (0.0%)	1 (1.8%)	
Mucositis oral			0.002
G0	12 (37.5%)	3 (5.5%)	
G1	16 (50.0%)	40 (72.7%)	
G2	3 (9.4%)	10 (18.2%)	
G3	1 (3.1%)	2 (3.6%)	

A total of 48 cases (55.2%) exhibited grade ≥ 3 late radiation injuries, of which 12 cases (37.5%) were reported in the N group and 36 cases (65.5%) in the CRT group. A total of 34 cases (39.1%) exhibited nasopharyngeal necrosis, of which 18 cases (20.7%) presented with necrosis of the internal carotid artery or other vessels, eventually leading to nasopharyngeal hemorrhage. A total of 19 cases (21.8%) presented with temporal lobe necrosis, whereas 16 cases (18.4%) exhibited cranial nerve injury, including 3 cases of facial nerve injury, 4 cases of optic nerve injury, 1 case of actinic nerve injury, 3 cases of auditory nerve injury, 7 cases of vagus nerve injury, 3 cases of glossopharyngeal nerve injury, 1 case of olfactory nerve injury and 2 cases of trigeminal nerve injury (> 1 type of cranial nerve injury may have occurred in each patient). A total of 7 cases (8.0%) presented with trismus (≤1 cm). The incidence rate of each late radiation injury was lower in the N group compared with that in the CRT group (except for trismus), and the overall incidence of late radiation injuries was significantly lower in the N group than that noted in the CRT group (*P* = 0.011) (Table 4).

**Table 4:** Grade ≥ 3 late radiation injuries in 87 patients of recurrent NPC

Complication	Median time of occurrence (months)	N Group	CRT Group	P
Nasopharyngeal necrosis	3.1 (0–55.7)	10 (31.3%)	24 (43.6%)	0.254
Hemorrhage	11.0 (3.7–58.5)	5 (15.6%)	13 (23.6%)	0.374
Temporal lobe necrosis	12.3 (0.5–35.1)	4 (12.5%)	15 (27.3%)	0.108
Cranial nerve palsy	NA	3 (9.4%)	13 (23.6%)	0.098
Trismus (≤1 cm)	NA	3 (9.4%)	4 (7.3%)	0.705
Total	–	12 (37.5%)	36 (65.5%)	0.011

*NA* symptoms were subjective and difficult to record

### Prognosis

Univariate analysis indicated that recurrent T classification, tumor response following RT and GTV-T volume were associated with OS. GTV-T volume was also associated with PFS, whereas plasma EBV DNA following RT was associated with DMFS. However, these factors were not significantly correlated with LRFFS (Table 5).

**Table 5:** Univariate analysis of potential prognostic factors

W	OS		PFS		DMFS	
	HR (95%CI)	P	HR (95%CI)	P	HR (95%CI)	P
Gender	0.74 (0.369–1.485)	0.397	0.849 (0.465–1.550)	0.595	1.70 (0.511–5.659)	0.387
Age (years)	1.713 (0.958–3.065)	0.07	1.441 (0.861–2.411)	0.164	0.641 (0.203–2.022)	0.448
Recurrent time	1.048 (0.588–1.867)	0.874	0.99 (0.586–1.671)	0.969	0.815 (0.258–2.570)	0.727
Recurrent T classification	3.716 (1.332–10.370)	0.012	2.005 (0.949–4.236)	0.068	1.531 (0.332–7.063)	0.595
Lymph node recurrence	0.727 (0.390–1.354)	0.315	0.699 (0.402–1.217)	0.206	1.378 (0.437–4.343)	0.585
EBV DNA prior to RT	1.061 (0.589–1.911)	0.843	1.199 (0.716–2.006)	0.491	1.245 (0.393–3.945)	0.709
EBV DNA following RT	1.563 (0.796–3.069)	0.195	1.664 (0.919–3.013)	0.092	3.294 (1.016–10.680)	0.047
Tumor response	1.791 (1.002–3.201)	0.049	1.455 (0.867–2.443)	0.155	0.681 (0.214–2.166)	0.515
Treatment modalities	1.103 (0.625–1.946)	0.736	0.908 (0.543–1.519)	0.713	0.871 (0.276–2.745)	0.813
GTV-T volume (cm^3^)	2.844 (1.539–5.257)	0.001	2.085 (1.224–3.553)	0.007	2.425 (0.714–8.230)	0.155
HB baseline	0.56 (0.313–1.001)	0.05	0.609 (0.361–1.027)	0.063	0.702 (0.209–2.352)	0.566

*HR* hazard ratio; *CI* confidence interval; *OS* overall survival; *PFS*, progression-free survival; *DMFS*, distant metastasis-free survival; *EBV*, Epstein-Barr virus; *HB*, hemoglobin. A Cox proportional hazards model was used to perform univariable analyses. All variables were transformed into categorical variables. HRs were calculated for gender (male vs. female); age (years) (< 50 vs. ≥50); recurrent time (< 36 months vs. ≥36 months); recurrent T classification (rT1–2 vs. rT3–4); lymph node recurrence (no vs. yes); EBV DNA prior to RT (negative vs. positive); EBV DNA following RT (negative vs. positive); tumor response (CR + PR vs. SD + PD); treatment modalities (chemotherapy vs. N); GTV-T volume (cm^3^) (< 30 vs. ≥30); and HB baseline (g/L) (< 135 g/L vs. ≥135 g/L).HR, hazard ratio; CI, confidence interval; OS, overall survival; PFS, progression-free survival; DMFS, distant metastasis-free survival; EBV, Epstein-Barr virus; HB, hemoglobin. A Cox proportional hazards model was used to perform univariable analyses. All variables were transformed into categorical variables. HRs were calculated for gender (male vs. female); age (years) (< 50 vs. ≥50); recurrent time (< 36 months vs. ≥36 months); recurrent T classification (rT1–2 vs. rT3–4); lymph node recurrence (no vs. yes); EBV DNA prior to RT (negative vs. positive); EBV DNA following RT (negative vs. positive); tumor response (CR + PR vs. SD + PD); treatment modalities (chemotherapy vs. N); GTV-T volume (cm^3^) (< 30 vs. ≥30); and HB baseline (g/L) (< 135 g/L vs. ≥135 g/L)

Multivariate analysis indicated that age ≥ 50 years and rT3–4 stage were independent prognostic factors affecting OS (HR = 2.369, *P* = 0.005; HR = 4.875, *P* = 0.003). OS was lower for patients aged ≥50 years compared with that for patients aged < 50 years (37.0% vs. 58.5%, respectively; *P* = 0.044). Moreover, OS was significantly lower for cases with rT3–4 stage compared with that noted for cases with rT1–2 stage (33.1% vs. 67.0%, respectively; *P* <  0.01). GTV-T volume ≥ 30 cm^3^ was an independent prognostic factor for PFS (HR = 2.031, *P* = 0.009). The latter was significantly lower for cases with GTV-T volume ≥ 30 cm^3^ compared with that noted for cases with GTV-T volume < 30 cm^3^ (17.0% vs. 35.2%, respectively; P <  0.01). Positive plasma EBV DNA following RT was an independent prognostic factor for DMFS (HR = 3.294, *P* = 0.047). DMFS was significantly lower in EBV DNA-positive cases following RT compared with that noted in negative cases (72.6% vs. 84.2%, respectively; *P* = 0.036). The use of chemotherapy or N was not a factor affecting prognosis (Table 6).

**Table 6:** Multivariate analysis of potential prognostic factors

Endpoint	Factor	P	HR (95%CI)
OS	Age (years)	0.005	2.369 (1.293–4.342)
	Recurrent T classification	0.003	4.875 (1.717–13.838)
PFS	GTV-T volume (cm3)	0.009	2.031 (1.190–3.467)
DMFS	EBV DNA following RT	0.047	3.294 (1.016–10.680)

*HR* hazard ratio; *CI* confidence interval; *OS* overall survival; *PFS* progression-free survival; *DMFS* distant metastasis-free survival; *EBV* Epstein-Barr virus. A Cox proportional hazards model was used to perform multivariate analyses. HRs were calculated for age (years) (**<** 50 vs. ≥50); recurrent T classification (rT1**–**2 vs. rT3**–**4); GTV-T volume (cm^**3**^) (**<** 30 vs. ≥30); and EBV DNA following RT (negative vs. positive)

### Toxicity-associated analysis

The chi-squared test indicated that the factors associated with severe late radiation injuries were recurrent T classification, tumor response following RT, GTV-T volume, treatment modality and low hemoglobin levels during RT. These five factors were included in the logistic regression analysis and the final model obtained was statistically significant (*P* <  0.001). The model was used to correctly classify 70.1% of the study subjects, with a model sensitivity specificity, positive predictive value and negative predictive value of 71.8, 68.8, 75.0 and 65.1%, respectively. The model results indicated that treatment modality and recurrent T classification were poor prognostic factors associated with severe late radiation injuries, with a 4.49-fold risk of grade ≥ 3 late radiation injury in the CRT group compared with that of the N group (*P* = 0.003). Moreover, a 9.06-fold risk of grade ≥ 3 late radiation injury was noted in the rT3–4 staging group compared with that of the rT1–2 staging group (P = 0.003).

## Discussion

The present study explored the long-term efficacy and toxicity profile of RT combined with N in patients with LR-NPC. A total of 87 patients were included for analysis with a median follow-up of 47.8 months. The results indicated that RT combined with N did not alter the 4-year OS, PFS, LRFFS and DMFS compared with CRT. In contrast to these observations, patients treated with N exhibited lower acute hematological toxicity and lower incidence of severe late radiation injuries. However, the survival rate of the patients in the present study was lower compared with that noted in a recent large meta-analysis that reported a 5-year OS of 41% [8]. The difference in survival rates may be due to the different proportion of patients with advanced disease compared with patients with locally recurrent advanced rT3-T4 accounting for 83.9% of all patients in the present study.

It has been shown that over 85% of patients with NPC overexpress EGFR [15]. EGFR is considered an important target in NPC therapy. N radiosensitizes cancer cells by inducing more apoptosis and unrepaired double strand breaks (DSBs) of DNA. The underlying mechanism of this radiosensitizing effect is related to the inhibition of DNA-PK involved DNA DSBs repair via the blockage of the PI3K-AKT pathway [16]. On the other hand, studies in vitro have shown that N binds bivalently (i.e., with both antibody arms to two targets simultaneously) to EGFR with moderate or high density, which is the stable pattern of attachment [17]. Therefore, N has demonstrated high safety profile and low toxicity and has become a research hotspot for tumor-targeted therapy due to its low affinity constant [12, 18, 19]. An increased number of studies have suggested that N with RT or chemotherapy has indicated promising efficacy without increasing toxicity for patients with cancer [14, 20–22]. The present study identified differences between the N and CRT groups with regard to acute hematological toxicity and severe late radiation injury. The grade range for leukopenia, neutropenia, thrombocytopenia and hepatic impairment was 0–1 in the N group, whereas the incidence of nausea, vomiting, dermatitis and oral mucositis was also reduced compared with that of the CRT group, suggesting that N exhibited an optimal safety and tolerability profile.

Following treatment of patients with LR-NPC with re-irradiation using IMRT, the incidence range of grade ≥ 3 advanced radiation injury was reported to be 0–74%, while the range of treatment-associated mortality (grade 5 toxicity) reached 0–65% [23]. In the present study, the incidence of grade ≥ 3 late radiation injury was 56.3%, whereas that of grade 5 late radiation injury was 29.9%, which was similar to the results of a recently reported meta-analysis [8]. A limited number of studies have explored the potential of chemotherapy to increase toxicity. The current study indicated that chemotherapy was the main factor causing grade ≥ 3 acute hematological toxicities. Severe late radiation injuries were more likely to occur in the CRT group compared with the N group (65.5% vs. 37.5%, respectively; *P* = 0.011). Although significant differences were not noted for each late radiation injury in the N group compared with the CRT group, the rates were lower and the incidence was significantly different from that of the CRT group, suggesting that N may reduce the occurrence of late radiation injuries compared with chemotherapy. Due to the specificity of LR-NPC and the retrospective nature of this study, nearly half of the patients were deceased at the time of follow-up and the Quality of Life questionnaire was difficult to implement. Therefore, we can only indirectly infer the quality of survival by reviewing the toxicities that occurred in patients during and after treatment. We still hypothesize that the quality of survival is improved in patients using N compared with those using chemotherapy, which may serve as potential treatment options in LR-NPC. However, larger clinical trials are required to verify our conclusion.

Yu et al. established a model and demonstrated that cumulative GTV dose ≥145.5 Gy, recurrent tumor volume ≥ 25.38 cm^3^, pre-irradiation necrosis and patient sex were factors associated with nasopharyngeal necrosis [24]. In contrast to these findings, the present study indicated that chemotherapy and recurrent T stage were independent prognostic factors for severe late radiation injury. Logistic regression analysis indicated that the risk of severe late radiation injury was 4.49 times higher in the CRT group compared with that in the N group (*P* = 0.003). Similarly, Su et al. reported a significantly higher rate of late toxicity in patients with LR-NPC receiving CRT compared with those treated with RT alone [25]. An additional factor, which was closely associated with the occurrence of toxicity, was recurrent T stage. Logistic regression analysis suggested a 9.06-fold risk of grade ≥ 3 late radiation injury for the rT3–4 stage group compared with that noted for the rT1–2 stage group (P = 0.003). This is consistent with clinical practice. Late disease stage is associated with higher invasion rate, higher radiation dose delivered to normal tissue, and higher incidence of toxicity.

Li et al. established a model and identified five important factors affecting the prognosis of LR-NPC as follows: age, recurrent GTV volume, recurrent T stage, previous RT grade ≥ 3 toxicity and re-irradiation dose (equivalent dose 2 value ≥68 Gy) [26]. The outcome of this study indicated that age and recurrent T stage were associated with OS, whereas GTV-T volume was associated with PFS. In the present study, re-irradiation dose, primary lesion + lymph node recurrence and recurrence time interval did not exhibit significant differences between the two groups, possibly due to the fact that the majority of the patients were irradiated at doses 60–66 Gy.

Due to the specificity of recurrent nasopharyngeal carcinoma, patients with early stage cancer usually receive surgery, while patients with advanced stage cancer usually receive chemoradiotherapy, and those who receive radiotherapy alone are usually treated palliatively. It is important to emphasize that most of the patients included in this study were at an advanced stage and were not suitable for surgery. Currently, there is no prospective clinical evidence to prove whether the addition of chemotherapy to re-RT is beneficial for recurrent NPC. Clinically, when chemotherapy is considered, clinicians prefer the sequence of induction with or without concurrent chemotherapy, based on the positive results from trials showing a survival benefit for locoregionally advanced primary NPC [27]. Absence of clinical studies, prior exposure to chemotherapy, and latency of recurrence and previous chemotherapy-related toxicity from the initial course should be considered in deciding the chemotherapy regimen. For these reasons, the patients in the CRT group included in present study were treated with different chemotherapy regimens, which still requires validation in further larger clinical trials.

In summary, RT combined with N achieved similar local control rates and OS for patients with LR-NPC compared with those reported for CRT. However, N exhibited lower acute toxicity and reduced incidence of late radiation injuries. It could also reduce the total treatment time of patients by subtracting induction chemotherapy. Since the present study was a retrospective analysis, selection bias could not be avoided and certain confounding factors may not have been considered in advance leading to missing data. Only single-center data were included in the present study, which resulted in insufficient sample size and short follow-up time period. Therefore, a higher sample size is required to verify these findings in future clinical studies.

## Conclusion

The treatment of LR-NPC is a clinical challenge that requires improved rate of local control, while minimizing toxic reactions. RT combined with N is an effective and selective strategy with comparable efficacy to CRT. This combination treatment method can also reduce part of the acute toxic reactions and severe late radiation injuries. Further prospective clinical studies with larger sample sizes are required to address this clinical challenge.

## Data Availability

The data that support the findings of this study are available on request from the corresponding author. The data are not publicly available due to privacy or ethical restrictions.
